# Supplementary material for: Altered cognitive processes shape tactile perception in autism
Source: eLife. 2026 Jun 10;14:RP108333. doi: 10.7554/eLife.108333 (PMC13252952; doi:10.7554/eLife.108333)
Supplement: Peer review history

# Editor report: eLife Assessment

**DOI:** 10.7554/eLife.108333.3.sa0

**Authors:** Jean-Paul Noel

**Affiliations:** University of Minnesota United States

**Keywords:** Convincing, Valuable

This study provides **valuable** insights with **convincing** evidence detailing altered tactile perception in a mouse model of ASD (Fmr1 mice), paralleling sensory abnormalities in Fragile X and autism. Its main strength lies in the use of a novel and quantitative tactile categorization task and the careful dissection of behavioral performance across training and difficulty levels, suggesting that deficits may stem from an interaction between sensory and cognitive processes. The behavioral experiments are well executed and set the stage for subsequent mechanistic, causal, and computational approaches. The work is relevant to those interested in autism, cognition, and/or sensory processing.

# Referee report: Reviewer #1 (Public review)

**DOI:** 10.7554/eLife.108333.3.sa1

[Editors' note: this version has been assessed by the Reviewing Editor without further input from the original reviewers. The authors have addressed the comments raised in the previous round of review.]

Summary:

This study addresses the important question of how top-down cognitive processes affect tactile perception in autism - specifically, in the Fmr1-/y genetic mouse model of autism. Using a 2AFC tactile task in behaving mice, the study investigated multiple aspects of perceptual processing, including perceptual learning, stimulus categorization and discrimination, as well as the influence of prior experience and attention.

Strengths:

The experiments seem well performed, with interesting results. Thus, this study can/will advance our understanding of atypical tactile perception and its relation to cognitive factors in autism.

# Referee report: Reviewer #2 (Public review)

**DOI:** 10.7554/eLife.108333.3.sa2

Summary:

This manuscript presents a tactile categorization task in head-fixed mice to test whether Fmr1 knockout mice display differences in vibrotactile discrimination using the forepaw. Tactile discrimination differences have been previously observed in humans with Fragile X Syndrome, autistic individuals, as well as mice with loss of Fmr1 across multiple studies. The authors show that during training, Fmr1 mutant mice display subtle deficits in perceptual learning of "low salience" stimuli, but not "high salience" stimuli, during the task. Following training, Fmr1 mutant mice displayed an enhanced tactile sensitivity under low-salience conditions but not high-salience stimulus conditions. The authors suggest that, under 'high cognitive load' conditions, Fmr1 mutant mouse performance during the lowest indentation stimuli presentations was affected, proposing an interplay of sensory and cognitive system disruptions that dynamically affect behavioral performance during the task.

Strengths:

The study employs a well-controlled vibrotactile discrimination task for head-fixed mice, which could serve as a platform for future mechanistic investigations. By examining performance across both training stages and stimulus "salience/difficulty" levels, the study provides a more nuanced view of how tactile processing deficits may emerge under different cognitive and sensory demands.

Weaknesses:

The study is primarily descriptive. The authors collect behavioral data and fit simple psychometric functions, but provide no neural recordings, causal manipulations, or computational modeling. Without mechanistic evidence, the conclusions remain speculative.

# Referee report: Reviewer #3 (Public review)

**DOI:** 10.7554/eLife.108333.3.sa3

Summary:

Developing consistent and reliable biomarkers is critically important for developing new pharmacological therapies in autism spectrum disorders (ASDs). Altered sensory perception is one of the hallmarks of autism and has been recently added to DSM-5 as one of the core symptoms of autism. Touch is one of the fundamental sensory modalities, yet it is currently understudied. Furthermore, there seems to be a discrepancy between different studies from different groups focusing on tactile discrimination. It is not clear if this discrepancy can be explained by different experimental setups, inconsistent terminology, or the heterogeneity of sensory processing alterations in ASDs. The authors aim to investigate the interplay between tactile discrimination and cognitive processes during perceptual decisions. They have developed a forepaw-based 2-alternative choice task for mice and investigated tactile perception and learning in Fmr1-/y mice

Strengths:

There are several strengths of this task: translational relevance to human psychophysical protocols, including controlled vibrotactile stimulation. In addition to the experimental setup, there are also several interesting findings: Fmr1-/y mice demonstrated choice consistency bias, which may result in impaired perceptual learning, and enhanced tactile discrimination in low-salience conditions, as well as attentional deficits with increased cognitive load. The increase in the error rates for low salience stimuli is interesting. These observations, together with the behavioral design, may have a promising translational potential and, if confirmed in humans, may be potentially used as biomarkers in ASD.

# Author comment: Author response

**DOI:** 10.7554/eLife.108333.3.sa4

**Authors:** Ourania Semelidou, Mathilde Tortochot-Megne Fotso, Adinda Winderickx, Andreas A Frick

**Affiliations:** Neurocentre Magendie Bordeaux France; Neurocentre Magendie Bordeaux France; Neurocentre Magendie Bordeaux France; Neurocentre Magendie Bordeaux France

The following is the authors’ response to the original reviews.

**eLife Assessment**

This study provides valuable insights with solid evidence into altered tactile perception in a mouse model of ASD (Fmr1 mice), paralleling sensory abnormalities in Fragile X and autism. Its main strength lies in the use of a novel tactile categorization task and the careful dissection of behavioral performance across training and difficulty levels, suggesting that deficits may stem from an interaction between sensory and cognitive processes. However, while the experiments are well executed, the reported effects are subtle and sometimes non-significant. The interpretation of results may be overextended given the nature of the data (solely behavioral), the reliance on repeated d′ measures may obfuscate some of the results without clearer psychometric or regressionbased analyses, and the absence of mechanistic, causal, or computational approaches limits the strength of the broader conclusions. The work will be relevant to those interested in autism, cognition, and/or sensory processing.

We thank the editors for their positive assessment of the data quality and the novelty of our behavioral task, and for pointing out the limitations inherent in behavioral studies.

We would like to clarify one important point regarding the use of d′ measures. While d′ was included to quantify sensitivity, our conclusions are not based solely on repeated d′ measures. In addition to d′, we analyzed raw behavioral data (correct and incorrect choice rates), and categorization performance was assessed using psychometric curves fitted with logistic regression models. These complementary analyses provide converging evidence and ensure that our interpretations are supported by multiple robust measures.

In the revised manuscript, we have further strengthened the analyses by including additional regression-based assessments, reporting effect sizes for subtle effects, and refining the statistical methods for clarity and transparency.

We fully acknowledge that this work is behavioral and does not directly reveal the underlying neural mechanisms. Nonetheless, the translational framework we have developed establishes a robust foundation for future studies. This platform can be directly applied in clinical research on autism and other neuropsychiatric conditions involving sensory-cognitive interactions, and provides a solid basis for subsequent mechanistic, causal, or computational investigations to uncover the neural circuits mediating these effects.

We greatly appreciate the editors’ and reviewers’ guidance and believe the revisions have clarified and strengthened the manuscript.

**Public Reviews:**

**Reviewer #1 (Public review):**

Summary:

This study addresses the important question of how top-down cognitive processes affect tactile perception in autism - specifically, in the Fmr1-/y genetic mouse model of autism. Using a 2AFC tactile task in behaving mice, the study investigated multiple aspects of perceptual processing, including perceptual learning, stimulus categorization and discrimination, as well as the influence of prior experience and attention.

We appreciate the reviewer’s statement highlighting the importance of our study.

Strengths:

The experiments seem well performed, with interesting results. Thus, this study can/will advance our understanding of atypical tactile perception and its relation to cognitive factors in autism.

We thank the reviewer for recognizing the quality of our experiments and the relevance of our findings for understanding tactile perception and cognition in autism.

Weaknesses:

Certain aspects of the analyses (and therefore the results) are unclear, which makes the manuscript difficult to understand. Clearer presentation, with the addition of more standard psychometric analyses, and/or other useful models (like logistic regression) would improve this aspect. The use of d' needs better explanation, both in terms of how and why these analyses are appropriate (and perhaps it should be applied for more specific needs rather than as a ubiquitous measure).

We thank the reviewer for these constructive comments. We acknowledge that aspects of the analyses were previously difficult to follow, and we have reworked the Results section to improve clarity and transparency.

We would like to emphasize that all d′ measures are complemented by analyses of raw response rates (correct and incorrect choices), ensuring that our interpretations are not solely dependent on this metric. In addition, we applied standard psychometric analyses wherever possible. For the training phase, only two stimulus amplitudes were presented, which precluded the construction of full psychometric curves; however, for the categorization phase, psychometric analyses were feasible and are reported in Figure 3. Specifically, psychometric functions were fitted to the data using logistic regression, allowing us to estimate both categorization bias (threshold) and precision (slope) across stimulus intensities. These analyses revealed no evidence of categorization bias or precision in *Fmr1*^-/y^ mice across stimulus strengths.

Following the reviewer’s suggestion, we have also added general linear model analyses that account for trial history, providing a complementary perspective on decision-making dynamics. Finally, while the calculation of d′ is detailed in the Methods, we have revised the Results to clearly explain its use and appropriateness in each relevant analysis.

These revisions aim to provide a clearer, more comprehensive picture of the data while ensuring that all conclusions are supported by multiple complementary measures.

**Reviewer #2 (Public review):**

Summary:

This manuscript presents a tactile categorization task in head-fixed mice to test whether Fmr1 knockout mice display differences in vibrotactile discrimination using the forepaw. Tactile discrimination differences have been previously observed in humans with Fragile X Syndrome, autistic individuals, as well as mice with loss of Fmr1 across multiple studies. The authors show that during training, Fmr1 mutant mice display subtle deficits in perceptual learning of "low salience" stimuli, but not "high salience" stimuli, during the task. Following training, Fmr1 mutant mice displayed an enhanced tactile sensitivity under low-salience conditions but not high-salience stimulus conditions. The authors suggest that, under 'high cognitive load' conditions, Fmr1 mutant mouse performance during the lowest indentation stimuli presentations was affected, proposing an interplay of sensory and cognitive system disruptions that dynamically affect behavioral performance during the task.

Strengths:

The study employs a well-controlled vibrotactile discrimination task for head-fixed mice, which could serve as a platform for future mechanistic investigations. By examining performance across both training stages and stimulus "salience/difficulty" levels, the study provides a more nuanced view of how tactile processing deficits may emerge under different cognitive and sensory demands.

We thank the reviewer for emphasizing the strengths of our task design and analysis approach, and we appreciate that the potential of this platform for future mechanistic investigations is recognized.

Weaknesses:

The study is primarily descriptive. The authors collect behavioral data and fit simple psychometric functions, but provide no neural recordings, causal manipulations, or computational modeling. Without mechanistic evidence, the conclusions remain speculative.

We thank the reviewer for the careful reading of our manuscript and for these constructive comments. We agree that our study is purely behavioral, and we appreciate the opportunity to clarify the scope and interpretation of our findings. The primary goal of this work was to characterize behavioral patterns during tactile discrimination and categorization in a translationally relevant mouse model of autism.

Although we did not include direct neural recordings, causal manipulations, or computational modeling, our analyses combining choice behavior, sensitivity measures from signal detection theory, psychometric curves, and regression-based models of trial history provide a detailed and robust characterization of perceptual learning, stimulus discrimination, categorization, and the interplay of cognitive processes with tactile perception. The manuscript has been revised to explicitly state that our conclusions are behavioral, emphasizing that this work establishes a foundation for future studies aimed at elucidating the neural and circuit mechanisms underlying these sensory–cognitive interactions.

Second, the authors repeatedly make strong claims about "categorical priors," "attention deficits," and "choice biases," but these constructs are inferred indirectly from secondary behavioral measures. Many of the effects are based on non-significant trends, and alternative explanations (such as differences in motivation, fatigue, satiety, stereotyped licking, and/or reward valuation) are not considered.

Alternative explanations for our findings including differences in motivation, fatigue, satiety, stereotyped licking, or reward valuation were carefully considered. As described in the Methods, only testing sessions with >70% correct performance on the training stimuli (12 µm and 26 µm) were included, excluding sessions with reduced motivation, fatigue, satiety, or stereotyped licking that could confound performance on low- or high-salience stimuli.

Although differences in reward valuation could affect learning speed, we observed no genotype differences in training duration (Fig. 1B-D, Fig. S1C-D). Sessions with disengagement were analyzed only during epochs of active task performance (information added to the revised Methods section, lines 619-620). Reward-driven choice biases were unlikely, as no genotype differences were observed in categorization bias (Fig. 3F) and GLM analyses confirmed that previous reward outcome did not affect current choices (Fig. 4D).

Finally, altered reward valuation could increase miss rates. Elevated miss rates in *Fmr1*^-/y^ mice were restricted to the lowest-intensity stimulus (12 µm) under high cognitive load, demonstrating a salience- and context-specific effect inconsistent with generalized motivational or reward deficits. The Discussion has been updated to clarify these points and delimit the scope of our interpretations (lines 483-499).

Third, the mapping of the behavioral results onto high-level cognitive constructs is tenuous and overstated. The authors' interpretations suggest that they directly tested cognitive theories such as Load Theory, Adaptive Resonance Theory, or Weak Central Coherence. However, the experiments do not manipulate or measure variables that would allow such theories to be tested. More specific comments are included below.

This was not done intentionally. References to Load Theory were meant to provide conceptual inspiration for assessing attention in high cognitive load conditions during categorization, rather than to indicate a formal test. Moreover, we do not claim to have tested the Weak Central Coherence theory, although our results suggest reduced facilitation of across- category discrimination. Finally, we agree that citing Adaptive Resonance Theory, which is grounded in artificial neural network models, could be misleading, and we have revised the text accordingly.

(1) The authors employ a two-choice behavioral task to assess forepaw tactile sensitivity in Fmr1 knockout mice. The data provide an interesting behavioral observation, but it is a descriptive study. Without mechanistic experiments, it is difficult to draw any conclusions, especially regarding top-down or bottom-up pathway dysfunctions. While the task design is elegant, the data remain correlational and do not advance our mechanistic understanding of Fmr1-related sensory and/or cognitive alterations.

We thank the reviewer for this comment and agree that our study is purely behavioral and does not provide direct mechanistic evidence for top-down pathway dysfunction. In the first version of the manuscript, the term “top-down” was used at the behavioral level, referring to the influence of higher-order cognitive processes (e.g., categorization, attention, sensory and choice history integration) on tactile perception, rather than to imply specific neural circuits.

We acknowledge that identifying the neural pathways underlying these effects would require extensive mechanistic experiments, including identifying the specific top-down pathway that modulates the influence of categorization on discrimination without directly altering categorization itself and performing pathway-specific recordings and manipulations. Such work represents a substantial mechanistic research program beyond the scope of the present study.

To clarify that our study does not provide insights into the neural underpinnings of the studied behavioral processes, we have revised the manuscript, removing the term “top-down” or replacing it with “higher-order processes” where appropriate. We also explicitly noted that future work using neural recordings or causal manipulations will be needed to uncover the neural underpinnings of these behavioral phenomena (lines 508-510).

(2) The conclusions hinge on speculative inferences about "reduced top-down categorization influence" or "choice consistency bias," but no neural, circuit-level, or causal manipulations (e.g., optogenetics, pharmacology, targeted lesions, modeling) are used to support these claims. Without mechanistic data, the translational impact is limited.

We recognize that terms such as “reduced top-down categorization influence” and “choice consistency bias” are derived from behavioral observations. However, we respectfully note that these behavioral inferences are widely used in clinical studies to characterize cognitive tendencies (Soulières et al., 2007; Feigin et al., 2021) and are not inherently speculative.

The translational impact of our work lies in the development of a robust behavioral platform that allows precise dissection of tactile perception and cognitive influences in a manner directly comparable to clinical studies. While we agree that neural, circuit-level, or causal manipulations would provide valuable mechanistic insight, the current study establishes a foundational behavioral framework that can guide and inform future investigations into the underlying neurobiological substrates.

To ensure clarity, we have revised the manuscript throughout to explicitly indicate that all conclusions are based on behavioral measures and do not imply mechanistic evidence.

(3) Statistical analysis:

(a) Several central claims are based on "trends" rather than statistically significant effects (e.g., reduced task sensitivity, reduced across-category facilitation). Building major interpretive arguments on non-significant findings undermines confidence in the conclusions.

We chose to present both statistically significant effects and trends to ensure transparency and to highlight that commonly used aggregate measures, such as d′, can sometimes obscure meaningful underlying patterns. In the text, p-values between 0.05 and 0.1 are described as trends without over-interpreting their significance. To further support interpretation, we have now computed effect sizes (Hedges’ g) for all subtle effects. In the revised manuscript, all interpretations of non-significant effects have been reworded to avoid overstatement.

(b) The n number for both genotypes should be increased. In several experiments (e.g., Figure 1D, 2E), one animal appears to be an outlier. Considering the subtle differences between genotypes, such an outlier could affect the statistical results and subsequent interpretations.

The number of mice used per genotype is consistent with standard practices in behavioral studies of sensory processing. To complement statistical analyses and account for small sample sizes, we have calculated effect sizes (Hedges’ g) for all subtle or trend-level effects (p ≈ 0.05–0.1), providing a measure of effect magnitude independent of sample size.

As the reviewer correctly noted, no animals were excluded as outliers, since observed variability reflects true biological differences rather than experimental or technical errors. In the revised manuscript, we re-examined all datasets for potential outliers, and when identified, analyses were performed both with and without the data point. Any results sensitive to single animals are explicitly reported. This procedure is now detailed in the Methods section (lines 675-679).

(c) The large number of comparisons across salience levels, categories, and trial histories raises concern for false positives. The manuscript does not clearly state how multiple comparisons were controlled.

We thank the reviewer for highlighting this important point. To control for false positives arising from multiple comparisons, we applied the Bonferroni correction. This information has been added to the Methods section (line 682) to ensure transparency and reproducibility of all statistical tests.

(d) The data in Figure 5, shown as separate panels per indentation value, are analyzed separately as t-tests or Mann-Whitney tests. However, individual comparisons are inappropriate for this type of data, as these are repeated stimulus applications across a given session. The data should be analyzed together and post-hoc comparisons reported. Given the very subtle difference in miss rates across control and mutant mice for 'low-salience' stimulus trials, this is unlikely to be a statistically meaningful difference when analyzed using a more appropriate test.

We thank the reviewer for raising this point, as this was not done intentionally. In the revised manuscript, miss rates for high- and low-salience stimuli were reanalyzed using a mixedeffects linear model, which appropriately accounts for repeated measurements within sessions (Fig. 5; Results section: lines 320-340). This analysis confirmed that *Fmr1*^-/y^ mice exhibit increased miss rates specifically at the 12 µm amplitude, with the effect disappearing at higher low-salience amplitudes (18 µm). Post-hoc comparisons with Bonferroni correction revealed a strong trend for increased misses at 12 µm (T-test: t = -2.8437, p = 0.058, Hedge’s g = 1.23), while no significant differences were found at other amplitudes. The Methods section has been updated to detail this statistical approach for analyzing miss rates (lines 686687).

(4) Emphasis on theoretical models:

The paper leans heavily on theories such as Adaptive Resonance Theory, Load Theory of Attention, and Weak Central Coherence, but the data do not actually test these frameworks in a rigorous way. The discussion should be reframed to highlight the potential relevance of these frameworks while acknowledging that the current data do not allow them to be assessed.

As mentioned above, our goal was not to directly test theoretical frameworks such as Adaptive Resonance Theory, Load Theory of Attention, or Weak Central Coherence, but rather to provide a context for interpreting our behavioral findings. In the revised manuscript, we have removed references to the Load Theory from the Results section and reframed the Discussion to emphasize that our results are consistent with certain predictions from these cognitive theories, without implying that the experiments directly assessed them. This clarifies that the interpretations are based on observed behavioral patterns, while still acknowledging the potential relevance of these frameworks to better understand tactile perception and cognition in autism.

**Reviewer #3 (Public review):**

Summary:

Developing consistent and reliable biomarkers is critically important for developing new pharmacological therapies in autism spectrum disorders (ASDs). Altered sensory perception is one of the hallmarks of autism and has been recently added to DSM-5 as one of the core symptoms of autism. Touch is one of the fundamental sensory modalities, yet it is currently understudied. Furthermore, there seems to be a discrepancy between different studies from different groups focusing on tactile discrimination. It is not clear if this discrepancy can be explained by different experimental setups, inconsistent terminology, or the heterogeneity of sensory processing alterations in ASDs. The authors aim to investigate the interplay between tactile discrimination and cognitive processes during perceptual decisions. They have developed a forepaw-based 2-alternative choice task for mice and investigated tactile perception and learning in Fmr1-/y mice.

Strengths:

There are several strengths of this task: translational relevance to human psychophysical protocols, including controlled vibrotactile stimulation. In addition to the experimental setup, there are also several interesting findings: Fmr1-/y mice demonstrated choice consistency bias, which may result in impaired perceptual learning, and enhanced tactile discrimination in low-salience conditions, as well as attentional deficits with increased cognitive load. The increase in the error rates for low salience stimuli is interesting. These observations, together with the behavioral design, may have a promising translational potential and, if confirmed in humans, may be potentially used as biomarkers in ASD.

We appreciate the reviewer’s positive assessment regarding our study’s translational value and the importance of our behavioral findings.

Weaknesses:

Some weaknesses are related to the lack of the original raster plots and density plots of licks under different conditions, learning rate vs time, and evaluation of the learning rate at different stages of learning. Overall, these data would help to answer the question of whether there are differences in learning strategies or neural circuit compensation in Fmr1-/y mice. It is also not clear if reversal learning is impaired in Fmr1-/y mice.

We thank the reviewer for these helpful suggestions. We agree that visualizing behavioral patterns, such as raster and density plots of licks, as well as learning rate over time, provides additional insights into learning dynamics. In response, we have added these analyses to the revised manuscript (Fig. S1, Fig. S2), which illustrate both individual and group-level learning trajectories and trial-by-trial licking patterns.

There was no assessment of reversal learning in *Fmr1*^-/y^ mice in this study. While this is an interesting and important question, and is motivated by previous preclinical and clinical findings, it falls outside the scope of the current manuscript.

**Recommendations for the authors:**

**Reviewer #1 (Recommendations for the authors):**

Main Comments

(1) This study addresses the important question of how top-down cognitive processes affect tactile perception in autism - specifically, in the Fmr1-/y genetic mouse model of autism vs. WT controls. Using a 2AFC tactile task in behaving mice, the study investigated multiple aspects of perceptual processing, including perceptual learning, stimulus categorization and discrimination, as well as the influence of prior experience and attention. The experiments seem well performed, with interesting results. I found certain aspects of the analysis not clearly explained, which made it difficult at times to understand.

Please see specific details in the comments below.

(2) To measure sensitivity, the authors present many comparisons of d' - sometimes between pairs of stimuli (or sometimes even for a single stimulus level).

(a) Firstly, the calculation of d' for a single stimulus value is unclear (because the same proportion of high/low choices for a given stimulus can result from shifts in bias/criterion).

We agree with the reviewer that calculating d′ for a single stimulus conflates sensitivity with response bias/criterion differences. For this reason, the panels showing d′ for individual stimulus amplitudes during training (Fig. 1F and 1G in the original manuscript) have been removed from the manuscript.

In addition, we revised our d’ (Fig. 1E) and criterion calculations (Fig. 2A), treating the high amplitude stimuli as “signal” and low amplitude stimuli as “noise”, based on the Signal Detection Theory. The formulas used in the revised manuscript take into account correct responses during high amplitude stimuli and wrong responses during low amplitude stimuli to calculate the sensitivity and bias of the mice during discrimination in the training period.

Sensitivity (d′) is now computed as:

*d' = z(lick right|high amplitude stimulus) - z(lick right|low amplitude stimulus)*

and the criterion (c) as:

c = −1/2 × [z(*lick right* / *high amplitude*) + z(*lick right* / *low amplitude*)]

(b) Secondly, while calculating d' makes sense for comparing two stimulus levels (like in the training condition), in the test condition (with a spread of stimuli), this becomes a little tedious - at times difficult to follow and unclear.

I would have thought that sensitivity (at least for overall performance) would be better compared using data from all the stimuli - e.g. either using:

(i) the sigma of the psychometric curve (although the downside of that approach is that it ignores history effects), or

(ii) a logistic regression for the choices, given the stimuli, where the weights assigned to the stimulus magnitude indicate sensitivity (the advantage of that approach is that history effects, like the previous trials/choices can be used as regressors in the model). Accordingly, it can simultaneously also quantify the history effects. This could even be expanded to a GLMM (mixed effects for different mice).

We thank the reviewer for this very valuable feedback. Indeed, during the testing phase, we calculated sensitivity d’ to probe the overall categorization sensitivity (Fig. 3H).

(i) This analysis was only complementary to the psychometric curves (fitted on the rightward lick rate for each stimulus amplitude using a general linear model – Fig. 3A). As the reviewer proposes, we had calculated the sigma of the psychometric curve (Fig. 3G, slope) to assess categorization precision. Sensitivity calculations have also now been revised using the aforementioned formula *(d' = z(lick right|high amplitude stimulus) - z(lick right|low amplitude stimulus))*.

(ii) To incorporate history effects, we implemented generalized linear models (GLMs) with a binomial link function to predict high-salience licks (right-lick choices) based on the current stimulus, trial history, genotype, and their interactions. A main-effects model included current stimulus, previous stimulus, previous outcome, previous choice, and genotype, followed by interaction terms to assess genotype-specific modulation of history effects. These analyses are now presented in the new Figure 6.

The resulting coefficients are shown in Fig. 6A. As expected, decisions were primarily driven by current stimulus amplitude (Fig. 6A, B). Both genotypes displayed a tendency to repeat previous choices (Fig. 6A, C), while previous reward outcomes did not influence current choice (Fig. 6A, D). Notably, stimulus amplitude history showed genotype-specific effects: WT mice were negatively influenced by the previous stimulus, whereas *Fmr1*^-/y^ mice remained unaffected (Fig. 6A, E).

To clearly visualize these findings, we plotted psychometric curves and marginal effects accounting for current stimulus, previous choice, previous outcome, and previous stimulus (Fig. 6B-E). These analyses are now fully integrated into the Methods (lines 688-702), Results (Fig. 6, lines 341-369), and Discussion (lines 469-479) sections of the revised manuscript.

(3) I find some of the terminology used confusing/misleading:

(a)The term "Categorization thresholds" can be misleading - in psychometric curves, "thresholds" often refer to the sigma (SD) of the fitted curve used to measure sensitivity (inversely related). Here, I think that the meaning is in terms of the PSE/ criterion. Perhaps the terminology can be improved to prevent confusion on this matter. E.g., I think that here the authors mean a measure of bias/criterion/PSE or similar. Correct? Not really a perceptual "threshold".

We thank the reviewer for pointing this out. In our analysis, the term “threshold” referred to the inflection point (i.e., the midpoint parameter μ) of the fitted logistic psychometric function used to categorize high- versus low-amplitude stimuli. We termed it “threshold” in the categorization of high and low amplitude stimuli. We agree with the reviewer that we could also use the term “Categorization bias”. We originally opted to avoid this term, not to confuse the readers when referring to the criterion (signal detection theory) as “response bias”. However, seeing as the term “threshold” may be confusing as well, we adopted the term “Categorization bias” in the updated version of the manuscript (lines 282, 284, 637-638, 785, Fig. 3F).

(b) Similarly, I think that "Categorization accuracy" can be misleading when describing the slope of the psychometric curve. Performance could have a steep slope but still be quite inaccurate (e.g., if there is a big bias). Perhaps "precision" is a better description of the slope?

We thank the reviewer for this suggestion. The slope of the psychometric curve is often referred to as “sensitivity” in the literature (Carandini and Churchland, 2014), but in our original manuscript we used the term “accuracy” to avoid confusion with the d′ measure from signal detection theory. We have revised the manuscript and Figures with the term “precision” as the reviewer suggested (lines 282, 284, 637-638, 786, Fig. 3G).

Minor Comments

(1) Abstract: "determines how autistic individuals engage" - there are other factors too. So, I think that "determines" is a little strong. Perhaps "influences" is more appropriate.

We have incorporated the reviewer’s suggestion (line 7).

(2) Figure 1 F, G. On the one hand, d' is defined as "sensitivity (d') in discriminating between high- and low-salience stimuli" - that seems to make sense. But then d' is also calculated and presented for each salience level on its own. How was this done? Namely, percent correct (or proportion of choices high/low salience) could be affected by criterion shifts as well as sensitivity. This makes calculating the d' for a single (low or high) salience stimulus ambiguous. So, how do these authors make this conclusion?

We agree that calculating d′ for a single stimulus amplitude is ambiguous, because the resulting value conflates true stimulus sensitivity with shifts in response bias or criterion. Consequently, all analyses and figures reporting d′ for individual high- or low-salience stimuli (e.g., Figures 1F and 1G) have been removed from the revised manuscript.

In the updated analyses, d′ is calculated only across high- versus low-salience stimuli, following standard Signal Detection Theory procedures, ensuring that it reflects true discriminability between the two categories (Methods, line 631; Figure 1E).

(3) "Our results showed comparable correct choice rates in Fmr1-/y and WT mice (Fig. 1H), for both high- and low-salience stimuli (Fig. S1C-D). In contrast, Fmr1-/y mice presented a significantly higher rate of incorrect choices (Fig. 1I)." - aren't correct choices and incorrect choices complementary (i.e., 1-x) in a 2AFC? How is this possible?

We thank the reviewer for pointing this out. Correct and incorrect choices are complementary at the single-trial level if miss trials are excluded. However, in our analyses, correct and incorrect choice rates were calculated by normalizing the number of correct or incorrect responses to the total number of trials (including misses), which breaks this complementarity and contributes to the differences observed in Fig. 1H–I. This was clarified in the Methods section (lines 616-617). Moreover, incorrect responses were less frequent than correct ones and are thought to reflect lapses, response bias, and impulsive responding rather than sensory performance, making them more sensitive to genotype-dependent differences in behavioral control. Based on this concept, we further examined whether incorrect choices were preferentially associated with specific stimulus amplitudes and assessed response bias and prior effects.

(4) The conclusion that "they showed a strong trend toward reduced sensitivity for lowsalience stimuli (Fig. 1G)" has a confound - it could be that there was a criterion shift (rather than differences in sensitivity)?

We agree with the reviewer that the previously reported trend in sensitivity for low-salience stimuli could reflect a criterion shift rather than true differences in sensory sensitivity. Because sensitivity estimates for individual stimulus amplitudes are not well-defined in a 2AFC framework, we have removed the sensitivity calculations for high- and low-salience stimuli considered independently. Instead, we now present salience-specific differences using correct and incorrect response rates for each stimulus amplitude**,** which more directly capture performance differences without assuming changes in sensory sensitivity (Fig. 1G-I, S1E-F).

(5) Figure 3D, E - I stumbled over this in comparison to Figure 3B, C. That is because (a) In D and E, the authors compare right-lick responses (reporting high salience) to stimuli of 12 μm and 14 μm amplitude (Figure 3D) and low-salience lick rates for the same (Figure 3E). I would have thought that these approaches are simply complementary (1-x) - see related minor question above/below. So, what is the advantage of presenting them both?

We presented both panels to clarify the source of the observed differences in performance. Specifically, showing right-lick responses (reporting high-salience choices) alongside low salience lick rates allows us to distinguish whether reduced high-salience reporting arises from an actual shift in choice (e.g., increased leftward licking) versus an increase in miss trials at the lowest amplitude (12 µm). By presenting both, we can demonstrate that the effect is primarily driven by an increase in leftward choices rather than by missed responses, providing a more precise interpretation of behavioral changes. The complementary analysis for leftward choices has now been moved to the supplemental material (Fig. S5A) and the reason for this analysis has been clarified in the Results (lines 275-276).

(b) In B and C, the authors compare two differences in stimulus magnitude (2 and 4 μm), but in Figure 3D and E, only one difference (2 μm) from two perspectives. I was expecting a comparison with stimuli differing by 4 μm in amplitude (comparable to the high stimulus comparison of 26 μm vs. 22 μm stimuli).

We have indeed analyzed the 12 μm versus 16 μm stimulus pair, which corresponds to a 4 μm difference and is reliably discriminated by both genotypes. In the original manuscript, we did not include this comparison because of the differences already seen at a 2 μm amplitude difference. Based on the reviewer’s suggestion, we have now included the 12 μm vs. 16 μm comparison in the revised manuscript (Results, lines 270-272; Fig. 3E) to provide a complementary perspective consistent with the high-salience comparisons (26 μm vs. 22 μm).

(c) "Sensitivity d' for high- and low-salience stimuli was calculated based on the Correct and Incorrect choice rate for high- and low-salience stimuli respectively." How were trials for which the animal did not respond taken into account? Were these part of the denominator? Or were these excluded when calculating proportions? (related to the Q regarding Figure 3 D,E above).

Indeed, the Miss trials were part of the denominator. This is now clarified in the Methods section (line 631).

(d) "c = d'(high)- d'(low)." - I did not understand this fully. There were several high and several slow stimuli - so how were these calculated? Pooled for high and pooled for low? Per stimulus difference?

This was indeed calculated for pooled high and low amplitudes during testing. In the revised manuscript, criterion c has been recalculated based on the average correct high rate (for stimuli of 20-26 µm amplitude) and average incorrect low rate (for stimuli of 12-18 µm amplitude), using the same formula as in the analysis of the training dataset:

c = −1/2 × [z(*lick right* / *high amplitude*) + z(*lick right* / *low amplitude*)]

Pooling across amplitudes allows us to obtain a single summary measure of response bias toward the right lickport, independent of stimulus discriminability. This approach is consistent with standard signal detection theory practices when multiple stimulus levels are present.

If the inter-trial interval is 5-10s, how is a 5s timeout a punishment?

The 5 s timeout serves as a punishment by temporarily delaying access to the next trial and potential reward, thereby reducing the overall reward rate. Even though the inter-trial interval (ITI) varies between 5 and 10 s, the timeout increases the effective delay before the next opportunity to earn a reward, discouraging incorrect responses. This is consistent with standard operant conditioning procedures, where brief timeouts act as negative consequences without being overly severe. Across most trials, the timeout effectively reduces expected reward rate, though its impact is minimal when the ITI is already long.

**Reviewer #2 (Recommendations for the authors):**

Task-related questions:

(1) What evidence is there that the 40 Hz, 12 μm stimulus is "low salience: while the 40 Hz, 26 μm stimulus is "high salience"? This seems like an arbitrary distinction without showing sensitivity curves across a group of animals. Better definitions of the stimuli and the actual forces applied are necessary.

We thank the reviewer for this comment. Based on our previous work (Semelidou et al., bioRxiv; Accepted in Advanced Science), both the 40 Hz, 12 µm and 40 Hz, 26 µm stimuli are clearly suprathreshold. In the present study, however, stimulus salience is defined in a relative and operational manner within this suprathreshold range.

Specifically, analysis of miss trials (Fig. S3E) shows that the 40 Hz, 12 μm stimulus consistently elicited a higher proportion of missed responses compared to the 40 Hz, 26 μm stimulus across animals, indicating lower behavioral performance for the lower-amplitude stimulus. We therefore refer to the 12 μm stimulus as “low salience” and the 26 μm stimulus as “high salience” to denote relative differences in perceptual strength and attentional engagement within the suprathreshold range, rather than differences in detectability or absolute sensory sensitivity. This definition has been clarified in the Methods (lines 583-587) and Results sections (lines 115-119; lines 225-227).

(2) Sensitivity curves/detection thresholds for each mouse should be included in the study.

We thank the reviewer for this suggestion. Sensitivity curves and detection thresholds for low-amplitude and low-frequency vibrotactile forepaw stimulation have been systematically characterized in our previous study (Semelidou et al., bioRxiv, Accepted in Advanced Science). In that work, we demonstrated that stimuli with similar amplitudes and even lower frequency (10Hz) than those used in the present study are reliably detectable by mice, confirming that both the 40 Hz, 12 µm and 40 Hz, 26 µm stimuli fall within the suprathreshold range**.**

Because the goal of the present study was not to determine absolute detection thresholds but rather to examine discrimination and categorization performance within a suprathreshold range, we did not re-establish full psychometric detection curves for each mouse.

We have clarified this rationale in the revised manuscript (Results, lines 108-113; Methods, lines: 577-579).

(3) What force is being applied during stimulus presentations? 12 or 26 μm does not provide enough information about the stimuli applied. What are the physical parameters of the indenter? What material, what tip size?

Vibrotactile stimuli were delivered to the forepaw via a piezoelectric actuator. A 12.7 mm stainless steel post (ThorLabs) was mounted on the actuator vertically and a 0.6 mm stainless steel rod (ThorLabs) was clamped horizontally onto this post. The horizontal rod served as the contact bar on which the animal rested its right forepaw.

Stimuli were sinusoidal vibrations at 40 Hz with peak-to-peak displacements of 12 μm (low salience) or 26 μm (high salience). The actuator displacement was calibrated prior to experiments to ensure accurate vibration amplitudes.

Animals were positioned in the setup to ensure stable and consistent forepaw contact with the rod delivering the vibration. Pilot experiments with an extra sensor to monitor forepaw placement confirmed that the mice did not remove their forepaws from the bar before stimulus delivery. All this information is now added in the Methods section (lines 552-555, 580-582).

(4) Only one vibration stimulus was used (40 Hz) - this preferentially activates specific subsets of low-threshold mechanoreceptors and not others. A range of vibrotactile stimuli (with varying frequencies) would be more useful. From this limited range of stimuli, it is difficult to assess whether the findings would extrapolate to other types of stimuli.

We agree that using a single vibration frequency limits the generalization of our findings across the full range of mechanoreceptor subtypes and vibrotactile stimulus conditions. In the present study, we deliberately focused on amplitude discrimination within the flutter range (<50 Hz), as this frequency preferentially activates subsets of low-threshold mechanoreceptors relevant for flutter perception and is commonly used in clinical studies of tactile amplitude discrimination (Puts et al., 2014, 2017; Asaridou et al., 2022). By holding frequency constant and varying only amplitude, we were able to isolate amplitude-dependent perceptual and decision-making processes while minimizing frequency-dependent variability and to facilitate direct translational comparisons with human studies using similar flutter stimuli.

We acknowledge, however, that extending the paradigm to additional, high frequencies would help determine whether the observed effects generalize across mechanoreceptor channels. We have now added this point as a future direction in the Discussion section (lines 510-514).

(5) The methods indicate that during the implementation of the water-restriction protocol, mice had access to a solid water supplement in their home cage. How did they control for how much water supplement was consumed by each mouse before the testing sessions?

We thank the reviewer for raising this point. The solid water supplement was divided into premeasured individual portions, and each mouse received its allotted amount only after the daily training/testing session. Daily body weight measurements were used to monitor hydration and ensure that all animals maintained stable body weight. If necessary, supplemental water was adjusted to maintain animals within the approved weight range. This procedure is now described in the Methods section (line 567-571).

(6) A control version of the test, perhaps using a different sensory modality, would be useful for making conclusions.

We agree that testing other sensory modalities would provide a useful control for assessing the generalizability of the observed effects. However, in the present study, we intentionally focused on the tactile modality, as touch has been shown to play a critical role in autism across sexes and predict other core behavioral symptoms. This makes touch particularly relevant for investigating translational mechanisms in this model.

By specifically targeting tactile perception, we aimed to investigate the link between sensory discrimination, decision-making, and cognitive modulation within a modality that is strongly implicated in autism. Previous studies in autistic individuals have demonstrated similar interactions between cognitive processes and perceptual decision-making in the visual domain, suggesting that such effects may not be modality-specific. Nevertheless, extending this paradigm to additional sensory systems would be valuable to directly test whether comparable cognitive influences on perception generalize across modalities. We have now incorporated this perspective as a future direction in the Discussion section (lines 514-518).

**Reviewer #3 (Recommendations for the authors):**

There are several questions:

(1) It is important to show stimulus intensity-response curves representing tactile responses for both WT and Fmr1-/y mice.

We thank the reviewer for this important comment. Detection sensitivity curves for lowamplitude and low-frequency vibrotactile stimulation of the forepaw have been characterized in detail in our previous study (Semelidou et al., bioRxiv; now accepted in Advanced Science). In that work, we showed that stimuli at or above 8 µm amplitude and 10Hz frequency are reliably detected by both WT and *Fmr1*^-/y^ mice.

Based on these findings, the current study employed vibrotactile stimuli at a higher frequency (40 Hz) and amplitudes of 12 µm and above, ensuring that all stimuli were well within the suprathreshold range for both genotypes. This experimental choice was made to specifically probe discrimination, categorization, and decision-making processes, rather than basic sensory detection. As a result, the behavioral effects reported here cannot be attributed to differences in stimulus detectability.

We have clarified this rationale in the revised manuscript to make explicit that the absence of full intensity-response curves in the current study reflects a deliberate focus on suprathreshold perceptual and cognitive processes rather than sensory threshold differences (Results, lines 108-113; Methods, lines: 577-579).

(2) There is no difference in the time it takes to learn the task between WT and Fmr1-/y mice. But how does the learning rate curve look? Is there a difference in the slope between WT and Fmr1-/y early vs late into learning?

We thank the reviewer for this suggestion. To directly address whether learning dynamics differed between genotypes, we analyzed learning curves across training.

We first computed the correct choice rate per day for each animal (Fig. S2A) and fit a mixedeffects model including training day, genotype, and their interaction. This analysis revealed no genotype differences in baseline performance or learning rate with minimal Genotype × Day interaction (Fig. S2A-top, Fig. S2C).

We additionally computed the slope of the learning curve for each individual, which also showed no difference across genotypes (Fig. S2B). In addition, within-animal day-to-day performance variability was also comparable across groups (Fig. S2A-bottom, S2D).

These analyses indicate that WT and *Fmr1*^-/y^ mice exhibit similar learning trajectories during training. The learning curves are now included in Figure S2, described in the Results (lines 140–151) and detailed in the Methods (lines 644-658).

(3) It would be useful to see raster plots of licks for different trials and the corresponding lick density plots for early vs late trials.

We thank the reviewer for this suggestion. To visualize trial-by-trial behavior, we included example lick traces from an early 100-trial session and a late 100-trial session, alongside the corresponding raster plots of licks (Fig. S1A–B).

(4) Consistent with the first question, examples of intermediate learning stages would help gain more insight into how both WT and Fmr1-/y mice learn.

In line with the reviewer’s suggestion, we examined whether WT and *Fmr1*^-/y^ mice showed different performance during intermediate stages of learning. To this end, we defined the middle three days of the training period of each animal as the intermediate learning phase. We compared both the mean correct-choice rate and individual learning slopes across this interval. Statistical analyses revealed no significant genotype differences in either measure, indicating comparable performance and learning dynamics during the intermediate phase of training (lines 152-156).

(5) How does the learning rate change with increased cognitive load for both WT and Fmr1-/y mice?

We thank the reviewer for this question. While our experimental design did not include a manipulation of cognitive load during the learning phase itself, we assessed whether increased cognitive load affected performance by analyzing behavior on the first day of testing, when animals were required to categorize and discriminate among a larger set of stimuli compared to training.

Using performance on the training stimuli during this first testing session as a proxy, we found no significant difference between WT and *Fmr1*^-/y^ mice in correct choice rate (Author response image 1). This indicates that increased cognitive load did not differentially affect performance on familiar stimuli across genotypes at this stage.

Because this analysis does not reflect learning rate per se, but rather performance under increased task demands after learning had already occurred, we did not incorporate it into the main Results section. Instead, it is presented here to directly address the reviewer’s question.

**Author response image 1.** Correct choice rate for the 12 µm and 26 µm stimuli during the first day of testing when the cognitive load is high.

(6) How does the learning rate change if the sensory stimuli are more challenging for both WT and Fmr1-/y to detect?

We thank the reviewer for this question. In the present study, animals were deliberately trained using well-separated, suprathreshold low- and high-salience stimuli to ensure reliable stimulus detection and to avoid confounding learning rate with perceptual difficulty or discrimination limits.

A recent study (Heimburg et al., 2025) has shown that learning is slower when the difference between the two training stimuli is reduced. Based on these results, we would expect that decreasing the separation between low- and high-salience stimuli would similarly increase training duration for both WT and *Fmr1*^-/y^ mice, since our results do not indicate any discrimination or categorization deficits in the mouse model of autism. However, directly testing how stimulus difficulty modulates learning rate would require a dedicated manipulation of stimulus spacing during training and was beyond the scope of the current study.

**Editor's note:**

Should you choose to revise your manuscript, if you have not already done so, please include full statistical reporting including exact p-values wherever possible alongside the summary statistics (test statistic and df) and, where appropriate, 95% confidence intervals.

These should be reported for all key questions and not only when the p-value is less than 0.05 in the main manuscript.
